# Theoretical analysis of currency reform and national governance challenges during the early Joseon Dynasty: A game theory approach

**DOI:** 10.1371/journal.pone.0286335

**Published:** 2023-06-02

**Authors:** Guanyu Hou, Ningning Hu

**Affiliations:** 1 Business School, China University of Political Science and Law, Beijing, China; 2 Graduate school, Chinese Academy of Fiscal Sciences, Beijing, China; Hosei University: Hosei Daigaku, JAPAN

## Abstract

During the early period of the Joseon Dynasty, the government undertook currency reform at both central and local levels to promote currency exchange and restructure market order. Drawing on historical sources and utilizing game theory methodologies, this study examines the challenges of state governance and the dynamics of central-local relations during this era. The findings suggest that the establishment of the Joseon Dynasty’s governance system arose from the rulers’ deliberate decisions; however, it was also driven by the necessity to reconcile the development of productive forces with the superstructure. The study highlights the impact of the “official” issue on communication efficiency between central and local authorities, which contributed to the currency reform’s failure. Consequently, the central government’s regulation and control over local regions, as well as its ability to govern the aspirations of grassroots populations, emerged as crucial factors for successful national governance. This research provides valuable insights into the academic value and significance of historical state governance practices and informs contemporary centrallocal relations and policy development.

## 1 Introduction

Preserving governmental resilience and transforming tensions and contradictions between central and local authorities into governance momentum are vital challenges confronting contemporary national governance. The Chinese experience in managing central-local relations has been disseminated across East Asian countries from an early stage, with varying degrees of divergence in implementation and exploration among these nations. In this context, the present study aims to explore the historical dynamics of central-local relations in the traditional Joseon Dynasty, which has had a profound influence on North Korea’s national governance model, revealing marked similarities with China’s system. By examining the central-local government relationships in the Joseon Dynasty, this research seeks to provide valuable insights and historical context for China’s ongoing national governance and deepening reforms.

Investigating policy information transmission and the central-local relationship in the traditional period, and utilizing history as a mirror, holds significant practical implications for contemporary academic research, theoretical discourse, policy development, and resource allocation [1]. This study, therefore, serves not only to illuminate the historical context of central-local relations but also to contribute to a broader understanding of the academic value and significance of such investigations in modern governance practices.

From a historical standpoint, expanding the scope of contemporary Chinese state governance and engaging in theoretical reflection is essential for understanding the central-local government relationship and addressing national governance issues. While research on China’s state governance predominantly concentrates on contemporary aspects, the country’s governance system exhibits a clear historical continuity that necessitates a more comprehensive exploration of its historical dimensions. By broadening the historical perspective, scholars can better contextualize and analyze the evolution of China’s state governance system and its implications for present-day challenges and opportunities [2].

To this end, this study delves into the monetary reform and state governance of the Joseon Dynasty during its traditional period by meticulously examining historical records and employing both static and dynamic game methodologies. The interaction between officials and the populace in the Joseon Dynasty’s currency reform highlights the central-local government relationship, suggesting that the hierarchical structure within modern bureaucracy, particularly the distribution of power across various levels, is crucial for the effective transmission of central directives and significantly influences the outcome of reforms. Contemporary central-local and civil affairs relations can also draw valuable insights from the Joseon Dynasty’s currency reform experiences to better inform decision-making and policy implementation.

## 2 Literature review

The formation, evolution, and implementation of currency are fundamentally predicated on state authority. National governance involves partial or comprehensive adjustments to the original production relations and superstructure, encompassing political, social, economic, cultural, and other domains. Monetary reform constitutes a crucial aspect of economic reform, targeting the stability and robustness of a nation’s financial system. This paper emphasizes the examination of the central-local government relationship during the Joseon Dynasty and offers a critical review of the pertinent research literature. Predominantly, existing studies have concentrated on the central-local government relationship, primarily investigating issues such as incentive structures and information asymmetry. Some scholars have explored the process from “central top-level design” to the “realization of institutional change,” providing a novel perspective for analyzing the financial interplay between central and local governments. By building upon these studies and incorporating the historical context of the Joseon Dynasty, this paper aims to contribute to the academic discourse on central-local government relationships and the broader implications for national governance and monetary reform [3]. Some scholars, such as Zhou, have identified that local officials exhibit dual characteristics as both “economic participants” and “political participants” when viewed through the lens of incentive structures. This dual nature highlights the complex roles that local officials play in the process of implementing monetary reforms and managing state governance [4]. Additionally, other researchers have approached the subject from the perspective of interpersonal relationships, employing the “principal-agent” model to analyze various aspects of intergovernmental relations and information asymmetry between central and local governments. Zhu Xufeng (2020) has noted that the transmission of policy information across different levels is susceptible to deviation and distortion. By understanding the challenges associated with information transmission and the complex roles of local officials, researchers can gain a more comprehensive understanding of central-local government dynamics, which in turn can inform strategies for improving policy implementation and overcoming information asymmetry [5].

Liu Peng (2016) argued that local preferences in China might become factors contributing to policy implementation deviations [6]. Chen Kang (2002) pointed out that in traditional society, constraints of transportation and communication, along with the costs of transmitting information on governance issues, varied across regions, as did the challenges they faced and their approaches to addressing them. The differing allocation of financial resources between central and local governments could lead to shifts in local behavior, with the central government’s financial power intensifying and transforming local governments from “aiding” to “grabbing” resources [7].

Shang Huping (2022) posited that the science and technology system in China integrated the country from bottom to top and vice versa. Following the Qin and Han dynasties, the establishment of local governments in China adopted the county system model’s vertical segmentation, with the primary distinction in specific operations manifested in the number of “layers” within the vertical structure [8].

Li et al. (2021) investigate the relationship between financial constraints, government subsidies, and corporate innovation in Chinese listed companies from 2007 to 2017. The authors’ findings emphasize the role of well-designed policies in driving economic development, a notion that can be paralleled to the currency reform in the early Joseon Dynasty. By employing advanced statistical methods, the study demonstrates the effectiveness of government interventions in mitigating financial constraints and stimulating innovation [9].

Similarly, Van Raan (2020) examines the impact of urban governance and local government structures on socio-economic urban scaling behavior in Denmark, the Netherlands, and Germany. The research suggests that urban areas governed by a single municipality perform better than those with fragmented governance structures. This finding provides valuable insights into the historical context of central-local relations and the role of governance structures in the success or failure of reforms, such as the currency reform in the early Joseon Dynasty, emphasizing the potential benefits of a unified governance approach in compact urban agglomerations [10].

Furthermore, the academic focus on “national governance” has experienced a subtle shift in recent years. Scholars have turned their attention to issues such as “power supervision,” “public crises,” “fundamental systems,” and “historical evolution.” Although existing studies have extensively investigated the economic, political, and cultural aspects of the Joseon Dynasty, research from the central government’s perspective remains limited. Hou Guanyu (2021) suggested that the slow economic development during the early Joseon Dynasty, policy incoherence, and the influence of international trade were the primary factors contributing to the failure of reforms in that period [11].

In summary, several studies have delved into central and local issues, focusing on perspective shifts, path selection, mode choice, and system construction. Utilizing empirical methods, existing research has proposed a range of concepts to encapsulate government behavior, offering fresh insights for examining the relationship between central and local governments. However, it is worth noting that only a limited number of studies have investigated the subject of monetary reform during the early days of the Joseon Dynasty from the central government’s perspective. Addressing this gap, the present paper adopts both static and dynamic game theory methodologies to explore the historical process of monetary reform in the early Joseon Dynasty from a central-local perspective. By doing so, this study aims to provide a valuable historical reference for the development and implementation of contemporary Chinese policies, enriching the academic discourse on central-local government relationships and the broader implications for national governance and monetary reform.

## 3 Establishing a national governance framework

In 1392, Lee Sung-Gyu established a new dynasty, and the following year, he proclaimed the country’s name as Joseon. Shortly after, the capital was relocated from Kaijing to Hanyang (present-day Seoul), marking the beginning of the historical era known as Lee Joseon. With the establishment of the Joseon Dynasty, a centralized rule centered around the king was further reinforced. The central government was composed of the Sizhiyuan, the Parliamentary Government, the Liu Cao, and the Three Divisions.

At the local level, the country was divided into eight provinces, each consisting of prefectures, sub-prefectures, counties, and sub-counties. Chiefs appointed by the king exercised administrative, judicial, and military powers at the local level. The implementation of the number plate law and the neighbor protection system allowed the Li Chao administration to closely monitor the population’s basic information and movement, exerting strict control over the local people’s freedom of movement. This historical context provides valuable insights into the role of central-local relations and governance structures in determining the success or failure of reforms, such as the currency reform in the early Joseon Dynasty.

Geographically, the Joseon Dynasty was unique as it was surrounded by the sea on its east and west, with the Yalu River forming the northwest border. The nation spanned 4,000 miles from east to west, with the capital situated 3,500 miles away. The country was administratively divided into eight provinces, each governed by an overseer. The establishment of the Joseon Dynasty maintained the decentralized power structure between central and local governments that had been implemented during the Goryeo period, with the central government responsible for managing both domestic and foreign affairs [12].

During the Taizong period, government affairs underwent restructuring, the distribution mechanism of state power was reshaped, and the organization of the central organs, as well as their powers and responsibilities, were clearly delineated. With the continuous improvement of the Korean government system, a national order of state control was established through the “Classics of the State.”

The Joseon Dynasty implemented a compulsory military service system, requiring farmers aged 16 to 60 (excluding certain classes such as enslaved people, monks, and butchers) to perform military service. To ensure an adequate supply of soldiers, the state implemented a military household system throughout the country. Those directly fulfilling military service obligations were designated as heads of households, while their supporters acted as guarantors or devotees. Under compulsory military service, the Joseon Dynasty maintained a standing army of 50,000 at the central level, 100,000 at the local level, 50,000 sailors, and 500 ships [13]. The king of the Joseon Dynasty, as the monarch representing the feudal ruling class, wielded significant power. The establishment of a “deliberative government” was divided into consular, left, and right deliberative governments, each with specific responsibilities and permissions. This historical context provides valuable insights into the role of central-local relations and governance structures in determining the success or failure of reforms, such as the currency reform in the early Joseon Dynasty (Table 1).

**Table 1:** Roles and responsibilities of key institutions in the Joseon Dynasty.

Institution Name	Duties and Responsibilities
Li Cao	Oversees appointment and dismissal of civil officials
Hu Cao	Manages tax collection and household registration
Li Cao	Handles etiquette and education matters
Bing Cao	Responsible for armed forces and defense against foreign aggression
Xing Cao	Addresses state and landlord property issues
Gong Cao	Construction, handicrafts, mountains and forests, rivers and lakes, etc

## 4 Construction and cost measurement of a governance system

This article focuses on the central-local relations within the Joseon Dynasty, particularly regarding the power dynamics between the central and local governments in governing economic and social affairs. The relationship between these two entities involves balancing the overall interests of the nation with local interests. The king of the Joseon Dynasty, as a monarch representing the feudal ruling class, held significant power. Rulers relied on a bureaucratic system of registration to exert control over local society.

The paper constructs a theoretical model that explores the relationship between the cost of supervision by rulers and the choice of institutions. This model demonstrates how the cost of supervision can influence changes at the official level. By analyzing the intricacies of central-local relations, the study sheds light on various factors that impact the effectiveness of governance during the Joseon Dynasty. It offers valuable insights into understanding the historical complexities of power dynamics and institutional arrangements [14].

In this analysis, the symbol Y represents the state output (tax revenue) of the Joseon Dynasty, while E denotes the degree of diligence of Joseon Dynasty officials. Assuming other conditions remain constant and considering the amount of social output as well as the rebellious tendencies of Joseon Dynasty officials, the income (tax revenue) of the Joseon Dynasty kings solely depended on the diligence of the officials. C represents the supervision costs incurred by the rulers of the Joseon Dynasty in overseeing the officials.

Let Y = F(E) and C = C(*β*, *γ*). Suppose *β* symbolizes the diligence of officials supervised by the rulers of the Joseon Dynasty, with *β* belonging to the interval (0, 1). Concurrently, *γ* represents the level of difficulty experienced by the Joseon Dynasty rulers in overseeing their officials:

$$\begin{array}{c} \gamma =\gamma (\text{A},\text{B},\text{C},\text{D},\text{Z},\text{Q}) \end{array}$$ (1)

Partial derivatives indicate the relationship between the degree of difficulty (*γ*) and the respective variables (A, B, C, D, Z, Q):

$$\begin{array}{c} \partial \gamma /\partial \text{A}>0,\partial \gamma /\partial \text{B}>0,\partial \gamma /\partial \text{C}>0,\partial \gamma /\partial \text{D}>0,\partial \gamma /\partial \text{Z}>0,\partial \gamma /\partial \text{Q}>0 \end{array}$$ (2)

These positive values imply that as any of these variables increase, the difficulty in supervision (*γ*) also increases.

*A* represents the territorial scope of the Joseon Dynasty and the challenges associated with its horizontal administration. Historical records state: “The Joseon Dynasty was bordered by the sea from east to west, the Yalu River in the northwest, and stretched 2,000 miles from east to west and 4,000 miles from north to south.” The territory was divided into eight provinces: Gyeonggi, Gyeongsang, Jeolla, Chungcheong, Hwanghae, Pyongan, Gangwon, and Hamgyong.

*B* represents the hierarchy of the Joseon Dynasty’s bureaucracy. The official system of the Joseon Dynasty was organized into two categories, comprising ranks from the first to the ninth, totaling eighteen distinct ranks. Among these, the third rank was further subdivided into upper and lower divisions. Ranks from the first to the upper third were referred to as Tang Chengguan officials. Ranks below the upper third to the seventh were called Tangxia Officials or Counselor Shangguan, and those below the seventh rank were considered Staff Officers.

*C* represents the complexity of national governance during the Joseon Dynasty. When officials transported goods, they often faced the threat of theft. For example, “Lu Song traveled to Kaizhou and encountered over 200 thieves, leading to the majority of the escorted horses being plundered.” Additionally, coastal areas were frequently invaded by Wokou pirates, causing disturbances and hardship for coastal residents. Furthermore, the misconduct of local officials contributed to the increase in administrative costs for the dynasty.

*D* represents the degree of collusion among officials. For instance, the exchange of gifts and bribes between officials was a prevalent issue, as they “mostly brought gold and silver native products to the Ming Dynasty to trade goods.” This practice further complicated the governance landscape and increased administrative challenges during the Joseon Dynasty [15].

*Z* represents the degree of close contact between the Joseon Dynasty and the Ming Dynasty, while *Q* signifies the level of closeness with other countries or regions. At the beginning of the 14th century, envoys from both countries maintained close exchanges and were frequently active in places such as Kaesong, Pyongyang, Uiju, and Donglai [13].

Based on the “Collection of Accounts of the Burning Chamber,” it is evident that “Although the villains’ names are Japanese, they are no different from Korea’s border people… Before the Rice Valley of Korea was obtained, the sons of the people could not bear to see their length and starved to death, and they all threw themselves into the water.” Relevant historical data suggests the following behavior of the rulers of the Joseon Dynasty:

$$\begin{array}{c} \text{Max}[\text{F}(\text{E})-\text{C}(\beta ,\gamma )] \end{array}$$ (3)

An authoritarian state can be described as an enterprise in which the ruler claims all the surplus (Bazel, 2001). In such a system, the bureaucracy cannot be motivated by surplus sharing, and thus the diligence of bureaucrats relies solely on the supervision exerted by the rulers of the Joseon Dynasty over their officials. Consequently, the first-order condition of the aforementioned formula is:

$$\begin{array}{c} {\text{F}}_{\text{E}}{\text{E}}_{\beta }-{\text{C}}_{\beta }=0 \end{array}$$ (4)

This implies that the optimal level of effort (supervision) that the rulers of the Joseon Dynasty were willing to exert needed to be satisfied. In other words, the benefits of increasing one unit of supervision would result in equal marginal returns to complete differentiation:

$$\begin{array}{c} ({\text{F}}_{\text{EE}}{{\text{E}}^{2}}_{\beta +}{\text{F}}_{\text{E}}{\text{E}}_{\beta \beta -}{\text{C}}_{\beta \beta })\text{d}\beta ={\text{C}}_{\beta \gamma }\text{d}\gamma \end{array}$$ (5)

$$\begin{array}{c} \text{d}\beta /\text{d}\gamma ={\text{C}}_{\beta \gamma }/({\text{F}}_{\text{EE}}{{\text{E}}^{2}}_{\beta +}{\text{F}}_{\text{E}}{\text{E}}_{\beta \beta -}{\text{C}}_{\beta \beta }) \end{array}$$ (6)

The denominator in the above equation represents the second-order condition for optimizing the level of supervisory effort by the rulers of the Joseon Dynasty. The value is less than 0 because, as the degree of supervisory difficulty *γ* increases, the rulers of the Joseon Dynasty intensify their supervision of the official group, which, in turn, results in an increase in marginal costs. Consequently, the numerators are greater than 0, leading to the following conclusion:

$$\begin{array}{c} \text{d}\beta /\text{d}\gamma <0 \end{array}$$ (7)

Therefore, under the assumption of maximization, the degree of supervisory efforts by the rulers of the Joseon Dynasty over their groups of officials was inversely proportional to the difficulty of supervision. With the country’s territorial size and bureaucratic level remaining constant, the complexity of grassroots people’s activities and the collusion among officials intensified, leading to an increase in the supervisory costs incurred by the rulers of the Joseon Dynasty. As a result, the rulers’ willingness to supervise declined marginally. Consequently, there is an urgent need to adjust the relationship between the central and local governments.

## 5 Gaming dynamics in the process of national governance

At the inception of the Joseon Dynasty, the currency system from the Goryeo period was adopted. The currency used during the Goryeo period can be categorized into two levels: upper and lower classes. The aristocratic class utilized silver bottles and fragmented silver, while ordinary grassroots people employed rice and cloth with a five-liter measurement. The traditional currency system became increasingly incompatible with the economic development of the Joseon Dynasty, reaching a critical point during the reign of Taejong of Joseon (1400-1418). Consequently, the rulers sought to further expand their fiscal authority.

**Fig 1:**
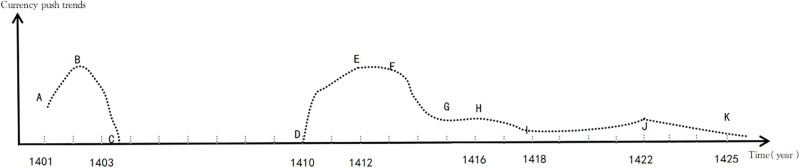
Trend chart of the monetary reform policies of the early Joseon Dynasty. Source: Adapted from the Records of the Joseon Dynasty, accessible at http://sillok.history.go.kr.

Depiction of the national governance process: Based on the pertinent historical sources on the currency reform of the Joseon Dynasty found in the Records of the Joseon Dynasty, and by plotting each point accordingly, a rough trend chart of the monetary reform policy during the early days of the Joseon Dynasty has been created. The horizontal axis represents time, while the vertical axis denotes the progression of monetary policy implementation. The segment A-C is considered the first stage of currency reform, whereas D-K represents the second phase of monetary reform (Fig 1 and Table 2).

**Table 2:** Roles and responsibilities of key institutions in the Joseon Dynasty.

Institution Name	Duties and Responsibilities
Li Cao	Oversees appointment and dismissal of civil officials
Hu Cao	Manages tax collection and household registration
Li Cao	Handles etiquette and education matters
Bing Cao	Responsible for armed forces and defense against foreign aggression
Xing Cao	Addresses state and landlord property issues
Gong Cao	Construction, handicrafts, mountains and forests, rivers and lakes, etc

In the fifth year of Sejong’s reign (1423), the people of the Joseon Dynasty engaged in extensive trade within the cities, with coins remaining as the primary circulating currency. It was noted that “although the law of the coin is indestructible, the intensity of trade during this period led to its widespread use among the people.” Sejong faced a dilemma between “enhancing the circulation credit of the coin” and “empathizing with the people’s livelihood.”

In the seventh year of Sejong’s reign (1425), a regulation was issued prohibiting the use of coins in the tribute of all households in the Joseon Dynasty. In the twenty-seventh year of Sejong’s reign (1445), selective use of coins was implemented. According to historical records, “The use of money varied across generations, and each country adhered to its own system. At the beginning, the use of goods was prevalent, and over the years, there were no significant drawbacks. Notably, the use of copper coins spanned generations, and it was a method of combined use of coins. However, as people’s opinions were divided, a ban on the use of coins was imposed, specifically on copper coins. However, since copper was not produced locally and was scarce, it was challenging to spend the funds, raising concerns. From then on, reusable goods were employed, and feasible conditions were listed later.”

Game behavior in monetary reform: The monetary reform process during the early years of the Joseon Dynasty was complex. As the primary policy-making body, the Joseon Dynasty government had to consider its interests while also taking into account the demands of different stakeholders. In the early days of the Joseon Dynasty, the implementation of monetary reform involved two primary players with opposing interests engaged in a game. One group comprised the “supporters,” or the officials of the Joseon Dynasty who advocated for monetary reform. The second group consisted of the “opponents,” or the grassroots people of the Joseon Dynasty who resisted currency reform. Both “supporters” and “opponents” aimed to maximize their respective interests. It is essential to note that during this period, the rulers of the Joseon Dynasty, specifically Taejong and Sejong, were considered third-party decision-makers who did not directly participate in the game between the “supporter” and “opponent” factions. Instead, they made “reasonable” decisions based on the game situation between the two parties.

Static Game Analysis: First, using the method of static game theory under complete information, we establish the following game hypothesis premises:

1. The currency reform officials and the non-reformist people, as the main game participants, align with the rational actor hypothesis in economics, making decisions based on their respective group interests.
2. There are two strategies of support and disapproval for the monetary reform plan proposed by Emperor Taejong of the Joseon Dynasty: supporting and agreeing with the government’s policies and measures and assisting in policy promotion, or not supporting the policy, expressed here as disapproval or adverse treatment of the policy.
3. Based on the analysis of historical materials from the Joseon Dynasty, the two interest groups involved in this game have clear interest demands. Assuming other factors remain unchanged, officials and the public actively participate in the game and express their interests through various means and methods, either directly or indirectly. The positions of the “supporters” and “opponents” in the game decision-making regarding currency reform implementation are apparent, satisfying the conditions for static game analysis.

Hypothesis: The mixed benefits of popular officials supporting the reforms (M11, G11); popular support, officials not supporting the mixed proceeds (M12, G12); the public not supporting but the mixed benefits when officials support (M21, G21); the mixed benefits when neither the public nor the officials support (M22, G22). The following yield matrix is obtained for the game analysis, as shown in Table 3 below.

**Table 3:** Payoff matrix for both players under complete information.

Joseon Dynasty Domestic People (M)	Joseon Dynasty Domestic Official (G)	
	Supported	Not Supported
**Supported**	(M11, G11)	(M12, G12)
**Not Supported**	(M21, G21)	(M22, G22)

In this game model, the grassroots people in the Joseon Dynasty represent the “non-reformist party,” and the domestic officials in the Joseon Dynasty represent the “reformist party.” In any scenario, the Joseon Dynasty’s official community aims to address historical issues inherited from the end of the Goryeo period by rectifying the currency system. Due to the monarchy, they could benefit more from supporting currency reform. Therefore, the benefits of choosing reform are higher for the official community; that is, G11 > G12 > G21 > G22. For the grassroots people accustomed to the original old currency and having a stable income, adopting the new currency would reduce people’s income, assuming other influencing factors remain unchanged. Consequently, the income for people not supporting the reform is typically higher than the income for those supporting the reform; that is, M12 < M22. However, if officials insist on reform, the Joseon Dynasty people “go with the flow” to benefit more; that is, M11 > M21.

In this analysis, we consider four possible strategy combinations for the “reformer” and “no reformer” sides. Let the probability of popular support for the currency reform in the Joseon Dynasty be PM1 = *ρ*, and the probability of not supporting the reform be PM2 = 1-*ρ*. Furthermore, let the probability of domestic officials supporting the reform be PG1 = *β*, and the probability of them not supporting the reform be PG2 = 1-*β*.

The expected utility for the domestic population can be expressed as:

$$\begin{array}{c} E(M)=\rho [M11\beta +(1-\beta )M12]+(1-\rho )[M21\beta +(1-\beta )M22] \end{array}$$ (8)

To analyze the game’s equilibrium, we derive the partial derivatives of E(M) with respect to *ρ* and *β*:

$$\begin{array}{c} \frac{\partial E(M)}{\partial \rho }=\left[M11\beta +\left(1-\beta \right)M12\right]-\left[M21\beta +\left(1-\beta \right)M22\right] \end{array}$$ (9)

$$\begin{array}{c} \frac{\partial E(M)}{\partial \beta }=\rho \left[M11-M12\right]+\left(1-\rho \right)\left[M21-M22\right] \end{array}$$ (10)

The equilibrium values for *β* and *ρ* can be calculated as:

$$\begin{array}{c} \beta^{*}=\frac{M12-M22}{\left(M11-M12)-(M21-M22\right)}\\ 1-\beta^{*}=\frac{M11-M12}{\left(M11-M12)-(M21-M22\right)}\\ \rho^{*}=\frac{M22-M21}{\left(M11-M21)-(M12-M22\right)}\\ 1-\rho^{*}=\frac{M11-M12}{\left(M11-M21)-(M12-M22\right)} \end{array}$$ (11)

Given that M12 < M22, β*, 1 − β*, ρ*, and 1 − ρ* all lie within the range (0,1), and M11 > M21, the Nash equilibrium of the complete information static game model is (support, support). This implies that both the public and officials will fully support the currency reform. Consequently, the Joseon Dynasty government, as a rational decision-maker, will choose to implement the currency reform based on the game’s outcome.

## 6 Dynamic game analysis

When Taejong of Joseon accepted the game’s outcome and implemented currency reforms, numerous challenges emerged in the dynasty [16]. Let R represent the benefits for the central government of the Joseon Dynasty, and E represent the degree of diligence of the officials of the Joseon Dynasty, with E1 denoting a high degree of diligence and E2 indicating low diligence. As mentioned earlier, assuming that the benefits of the Joseon Dynasty depend solely on the diligence of local officials, R(E1) > R(E2).

*C* represents the cost of supervising the officials by the rulers of the Joseon Dynasty, *β* denotes the degree of diligence of the Joseon Dynasty rulers in supervising the officials, with *β*_1_ signifying a high degree of supervision and *β*_2_ indicating low levels of supervision. Hence, *C*(*β*_1_) > *C*(*β*_2_). Meanwhile, *γ* signifies the difficulty for the rulers of Joseon to supervise officials, with *β*, *γ* ∈ (0, 1).

If the reform is successfully implemented, the central government will provide corresponding rewards to local officials, such as increasing the number of officials or offering promotions, with more diligent officials receiving higher rewards. Let *w* represent the rewards received, where *w*(*E*1) > *w*(*E*2). Simultaneously, if local officials do not support the ruler’s reforms, they may face punishment from the ruler, such as demotion or other penalties, denoted as *T*.

If Emperor Taizong did not implement monetary reforms, the combination of central and local benefits would be (0, 0). If Emperor Taizong chose to implement currency reforms, but local officials did not support them, the combination of benefits between the two sides would be (0, −*T*). If Emperor Taizong decided to carry out reforms and local officials complied with the central government’s orders, there would be two scenarios:

1. Local officials are more diligent and strive to carry out reforms, resulting in combined benefits of (*R*(*E*1) − *C*(*β*_1_, *γ*) − *w*(*E*1), *w*(*E*1) − *E*1).
2. Local officials are not diligent enough, leading to a combination of benefits between the two parties of (*R*(*E*2) − *C*(*β*_2_, *γ*) − *w*(*E*2), *w*(*E*2) − *E*2).

**Fig 2:**
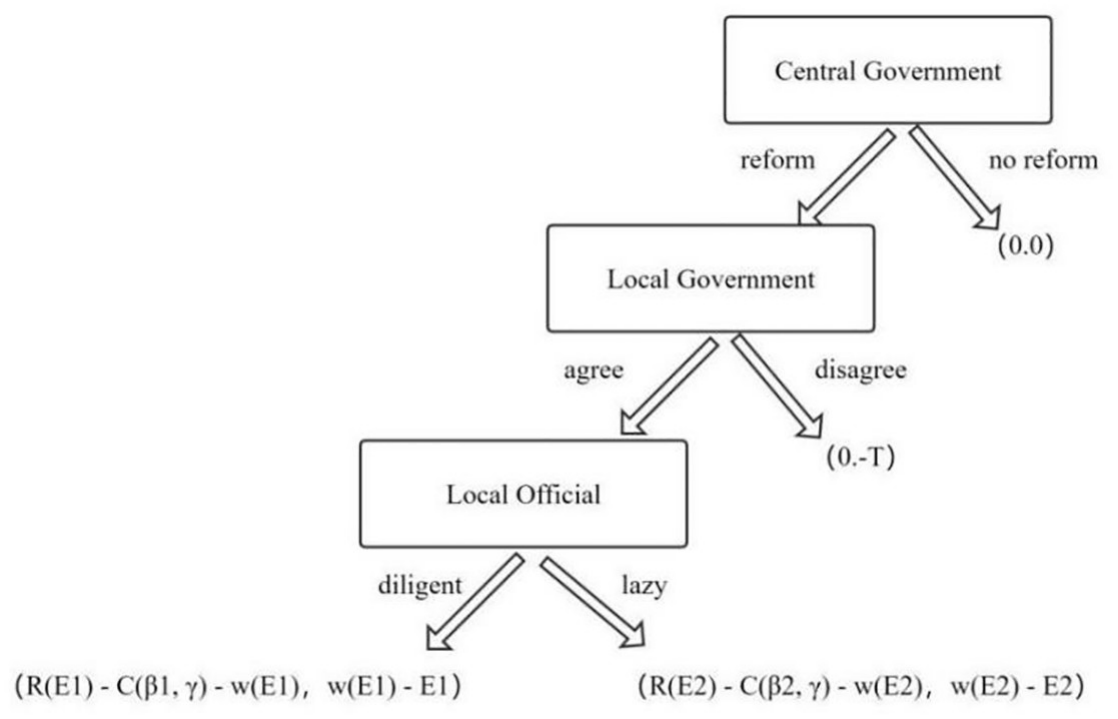
Dynamic game tree between central and local governments.

By combining historical data analysis, we can construct a dynamic game tree (Fig 2). Utilizing the backward induction method, we first analyze the choice of local officials in the third stage, “whether to work diligently,” in implementing reform policies. If w(E1) − E1 > w(E2) − E2, local officials will opt to work on the reform policies. Reverting to the second stage, local officials will choose to work hard on implementing reforms if w(E1) − E1 > −T, meaning that the net benefit of local officials from implementing reforms is greater than the penalty for not implementing them.

Returning to the first stage, the central government will choose to implement the reform strategy if R(E1) − C(*β*1, *γ*) − w(E1) > 0, indicating that the benefits of monetary reform for the central government outweigh the sum of its supervision costs and the rewards it pays to officials. In this scenario, the outcome is most favorable; the ruler’s reform policies will be fully supported and implemented by local officials. This, in turn, significantly increases the probability of popular support for the reform and optimizes the implementation of currency reform.

To achieve this, local officials who diligently implement reforms must be compensated more than those who are negligent, less the increase in the negative utility of their diligence over their inattention. Additionally, penalties imposed on officials who do not support reform should be severe enough. Moreover, the success of the central government in achieving results through monetary reform depends not only on the effectiveness of the policy itself but also on the magnitude of the central government’s supervision costs for local governments.

In summary, during the reign of Emperor Taizong of the Joseon Dynasty, efforts were made to implement specific policies based on information provided by local officials, with the aim of transitioning from cloth to paper currency. According to the formula C = C(*β*, *γ*), *γ*(A, B, C, D, Z, Q), the horizontal management structure of the Joseon Dynasty was highly complex, spanning from the central government to proximate and remote regions. Consequently, the Taizong administration faced challenges in collecting local information and experienced delays in policy implementation following the decision to pursue monetary reforms. As the policies were enacted, the diligence of officials progressively declined, leading to a decrease in public support. This, in turn, diminished the central government’s benefits, increased the costs of supervision, and ultimately resulted in the failure of the currency reforms. In order to effectively carry out monetary reforms, it is crucial to establish a proper balance in the relationship between the central and local governments.

## 7 Conclusions

The historical experience of the Central Korean Dynasty demonstrates that direct government management costs were high due to insufficient information transmission between regions. In certain periods, reform emerged as the most effective means to break the deadlock and optimize distribution. However, striking a balance between local development interests, including the rational allocation of power and interests, finding the greatest common denominator and equilibrium point, and maintaining central authority, was crucial in determining the success or failure of reform. During the traditional period, national governance primarily focused on the relationships and tensions between individuals and collectives, central and local governments, the state and society, and the transmission efficiency of information networks and information fields. The study found that:

1. The establishment of the Joseon Dynasty’s bureaucratic system might appear as a result of the rulers’ subjective choice, but it actually stemmed from the progress of the productive forces, which urgently required adjustments to the superstructure in line with their development direction.
2. Information transmission from the central to the local level involved many layers, leading to omissions and inaccuracies. The root cause of smooth or blocked information channels lay in “personnel.” Although traffic conditions and population size could impact information transmission efficiency, “the connection of channel levels” or “power hubs” often played a crucial role in information loss.
3. Monetary reform faced numerous challenges. Improving the country’s governance capacity was not an easy task; it necessitated adopting a systemic approach to consider the broader situation. Successful reform implementation required more than merely addressing economic issues or employing economic means. The high cost of formulating, implementing, and evaluating monetary policy in Li Chao was mainly reflected in the difficulty of gathering strategies and information required for implementing such policy.
4. Central, local, and individual governments calculated the expected costs and benefits following a system change, considering inherent norms and other opportunities. During monetary reform implementation, stakeholders under the original system often resisted and suppressed change, while those who had suffered losses consistently proposed strategies to promote change.

## 8 Historical experience

The relationship between officials and the people during the monetary reform of the Joseon Dynasty, as well as the challenges faced by the central government in managing central-local relations, continue to hold practical significance and offer insights for contemporary social development.

1. Emphasizing the government’s “control function” is indispensable in the reform process. Regulating grass-roots society’s intentions may prove crucial to the success of the reform. When implementing reforms, the central government should adopt a supervisory mechanism with clear rewards and punishments for local governments. High incentives and punishments significantly increase the likelihood of successful reform.
2. It is essential to establish reasonable central and local levels to reduce regulatory costs. Before implementing the reform, the central government should conduct thorough investigations, initiate partial pilots, and then implement the reform comprehensively to minimize hidden costs.
3. In the national governance process, formal norms are formulated and implemented through organizational behavior, standardizing and managing constraints on members while enhancing government efficiency through reward and punishment mechanisms. Informal systems, primarily based on grassroots individuals’ interactions, strengthen social networks and promote social governance. It is vital to recognize the importance of formal institutions while acknowledging the subtle influence of informal systems.
4. The system’s value and governance practices may become disconnected in actual operation. Although governance extends institutional values in practical terms, various factors influence the outcomes of governance activities. Consequently, the inadequacy of governance results cannot simply be attributed to an improper system value.
5. Strengthening communication and information flow between central and local governments is essential to improve policy implementation and decision-making. By investing in modern communication technologies and promoting a culture of transparency and collaboration, governments can minimize information loss, reduce misunderstandings, and foster more effective governance.
6. Engaging and incorporating local communities’ perspectives in the reform process can increase the likelihood of success. By actively seeking feedback, encouraging participation, and addressing local concerns, governments can build trust, improve public understanding, and ultimately achieve more sustainable and effective reforms that address the specific needs of the communities they serve.
